# Pathways Linking Health Literacy to Self-Management in People with Type 2 Diabetes

**DOI:** 10.3390/healthcare9121734

**Published:** 2021-12-15

**Authors:** Eun-Hyun Lee, Young Whee Lee, Duckhee Chae, Kwan-Woo Lee, Seongbin Hong, So Hun Kim, Jin Ook Chung

**Affiliations:** 1 Graduate School of Public Health, Ajou University, Suwon 16499, Korea; 2 Department of Nursing, Inha University, Incheon 22212, Korea; ywlee@inha.ac.kr; 3 College of Nursing, Chonnam National University, Gwangju 61469, Korea; dheechae@jnu.ac.kr; 4 Department of Endocrinology and Metabolism, School of Medicine, Ajou University, Suwon 16499, Korea; LKW65@ajou.ac.kr; 5 Department of Internal Medicine, School of Medicine, Inha University, Incheon 22212, Korea; sbhongmd@inha.ac.kr (S.H.); shoney@inha.ac.kr (S.H.K.); 6 Department of Internal Medicine, Chonnam National University Medical School, Gwangju 61469, Korea; imagine-jjo@hanmail.net

**Keywords:** diabetes, health literacy, mediation effect, self-efficacy, self-management, social isolation

## Abstract

Health literacy is considered to be an emerging determinant of health behaviors and outcomes. The underlying mechanisms linking health literacy to diabetes self-management are currently unclear. This study assessed a mediation model consisting of a direct pathway between health literacy and self-management, and indirect pathways via social isolation only, self-efficacy only, and social isolation and self-efficacy serially in people with type 2 diabetes. A cross-sectional design was employed, and a total of 524 participants were recruited from outpatient clinics of multi-institutions from June 2020 to February 2021. The mediation model was analyzed using the PROCESS macro on SPSS with bootstrap bias-corrected 95% confidence intervals (CIs) with 10,000 bootstrapping iterations. Health literacy positively affected self-management. The estimated indirect effect of health literacy on self-management via social isolation was significant, at 0.018 (95% CI = 0.004–0.036). The indirect effect via self-efficacy was estimated at 0.214 (95% CI = 0.165–0.266). The indirect effect via social isolation and self-efficacy serially was 0.013 (95% CI = 0.006–0.023). The findings of this study suggest that clinical practice can be improved through more comprehensive diabetes self-management interventions that promote all of the components of health literacy, social contacts/networks, and self-efficacy in particular.

## 1. Introduction

Diabetes is a global health problem. Approximately 463 million people had diabetes worldwide in 2019, and this number is estimated to reach 700 million by 2045 [1]. Type 2 diabetes accounts for about 90% of all diabetes cases [1]. These people need to perform ongoing self-management in their daily lives, such as physical exercise, healthy diets, emotional coping, taking medication, self-monitoring of blood glucose, symptom regulation, and foot inspections [2]. Self-management plays a pivotal role in successful treatments for improving metabolic control and the quality of life, and reducing the risk of complications and health-care costs [1]. It is therefore important for health professionals to identify factors that increase the risk of and to promote diabetes self-management in order to develop evidence-based interventions to improve self-management among people with type 2 diabetes.

Health literacy has been described as “cognitive and social skills that determine the motivation and ability of individuals to gain access to, understand, and use health information in ways that promote and maintain good health” [3]. Health literacy is considered an important determinant of health behaviors and outcomes [4], and is considered to be positively associated with self-management among people with diabetes [5,6]. However, the underlying mechanisms of this association are currently unclear [7]. Fransen et al. [8] suggested that it would be useful to identify sociocognitive mediators for these mechanisms.

According to social cognitive theory, self-efficacy is “the belief in one’s capability to organize and execute the course of action required to produce given levels of attainments” [9]. Self-efficacy was suggested to mediate health literacy and self-management in diabetes cases [7,8]. However, such empirical evidence is severely lacking for people with diabetes. A study involving 459 people with type 2 diabetes found that health literacy indirectly affected self-management via self-efficacy [10]. More specifically, self-efficacy mediated the relationship between health literacy (particularly numeracy) and diabetes medication adherence [11]. Considering this insufficient evidence, it was recommended to further investigate self-efficacy as a mediator using a systematic review study of health literacy and diabetes outcomes [12].

Squiers et al. [13] suggested that various mediators may affect the relationship between health literacy and health behaviors/outcomes, which might be specific to each behavior or outcome. Other researchers suggested that self-efficacy does not entirely explain how health literacy influences self-management [14,15]. This means that other potential mediators that considerably account for this pathway must be identified.

Social isolation may be a plausible mediator, which is a structural indicator of social connection that refers to an objective and quantitative measure of network size, diversity, and interpersonal contact frequency [16]. Social isolation needs particular attention during the novel coronavirus disease 2019 (COVID-19) pandemic where quarantine policies have been implemented to ensure social distancing, such as keeping 2 m between people and restricting gatherings, indoor exercise, and public transportation.

People with lower health literacy levels tended to be less interested in social engagement [17], and greater health literacy has been linked to favorable social contacts and lesser social isolation [18,19]. Social isolation has also been suggested to negatively influence health behaviors. This means that those without regular interpersonal contact and involvement in social organizations are likely to perform more adverse health behaviors, such as a poor diet or insufficient physical activity [20,21]. Similarly, a negative relationship has also been suggested between social isolation and diabetes self-management [22,23]. Health literacy can therefore by hypothesized to be indirectly related to self-management via social isolation as a mediator.

Social isolation was reported to be significantly correlated with self-efficacy in people with type 2 diabetes [24]. Wu and Sheng [25] noted that the closeness of social circles and frequency of interpersonal contact indirectly affected health behaviors (e.g., physical activity, nutrition, and stress management) via self-efficacy, which represents evidence that social isolation affects self-efficacy. Health literacy was hypothesized to be linked with self-management indirectly via social isolation and self-efficacy serially, which is considered a serial mediation effect [26], among people with type 2 diabetes.

Based on the literature review, the present study assessed a mediation model for people with type 2 diabetes that consisted of the following hypothesized pathways: a direct effect of health literacy on self-management, and three different indirect (mediation) effects via social isolation only, self-efficacy only, and both social isolation and self-efficacy serially.

## 2. Methods

### 2.1. Sample and Data Collection

This study had a cross-sectional design. A total of 524 participants were recruited from outpatient clinics of multiple institutes in the Republic of Korea from June 2020 to February 2021 using convenience sampling. The sample size satisfied the mediation model recommendation of 450–500 cases for a statistical power of 80% to validate indirect effects [27,28]. The inclusion criteria were being at least 19 years old, able to speak and write Korean, and participating in a treatment regimen for type 2 diabetes (oral hypoglycemic agents, insulin, or both). People diagnosed with gestational diabetes were excluded. Trained research assistants met potential participants at the outpatient clinics, and those who expressed an interest in participating were provided with information on the study. Those who agreed to participate were asked to sign a formal informed-consent form and to complete questionnaires.

### 2.2. Ethical Considerations

This study was approved by the institutional review boards of the hospitals from which the participants were recruited. The participants were provided with information about the purpose of this study, the voluntary nature of participation and the right to refuse to answer or withdraw, and risks and benefits of participation. All participants signed the informed-consent form and received remuneration.

### 2.3. Measures

#### 2.3.1. Health Literacy

The Diabetes Health Literacy Scale (DHLS) was used, which is a disease-specific health literacy instrument [29] that consists of 14 items scored on a five-point Likert scale in three subscales: informational (seven items), numerate (four items), and communicative health literacy (three items). The scale score is the average of these items, with higher scores indicating better health literacy. The DHLS exhibited good psychometric properties in tests of four validity (content, structural, convergent, and criterion) and two reliability (internal consistency and test–retest reliability) aspects. Cronbach’s alpha of the DHLS in the present study was 0.93.

#### 2.3.2. Social Isolation

Shankar et al. [16] developed a social isolation index based on five binary items asking whether a respondent (1) is not married/not living with a partner; (2) does not participate in any organization, religious group, or committee; and has interpersonal contact less than once per month (including face-to-face, telephone, or written/e-mail) with (3) friends, (4) relatives, and (5) other family members who they do not live with. Scores ranged from 0 to 5, with higher scores indicating greater social isolation.

#### 2.3.3. Self-Efficacy

Self-efficacy was determined by the four-factor, 16-item Korean version of the Diabetes Management Self-Efficacy Scale (K-DMSES) [30] derived from the original 20-item Diabetes Management Self-Efficacy Scale (DMSES) [31]. Total scores were calculated as the sum of scores for all items on this scale, with higher scores indicating greater self-efficacy in self-management. The K-DMSES demonstrated satisfactory content, factorial-construct, and concurrent validities, internal consistency (Cronbach’s alpha = 0.92), and test–retest reliability (intraclass correlation coefficient [ICC] = 0.85) [30]. Cronbach’s alpha of the K-DMSES in the present study was 0.94.

#### 2.3.4. Self-Management

Self-management was assessed using the Diabetes Self-Management Scale (DSMS), which is a new instrument that measures the complex nature of self-management among people with type 2 diabetes, and consists of 17 items scored on a five-point Likert scale [6]. The DSMS exhibited satisfactory psychometric properties for content, structural, and convergent validities, internal consistency (Cronbach’s alpha = 0.74–0.88), and test–retest reliability (ICC = 0.66–0.94) when tested on 473 people with type 2 diabetes [6]. Cronbach’s alpha of the DSMS in the present study was 0.91.

#### 2.3.5. Demographic and Medical Information

Questionnaires on gender, age, monthly income, education status, and diabetes education were administered. Diabetes education was assessed using the following yes/no question: “Have you ever received education about diabetes?” Medical information on treatment regimens, glycated hemoglobin (HbA1c) level, and the duration of diabetes were collected from the medical records of the participants.

### 2.4. Statistical Analyses

Data were analyzed using the PROCESS macro (version 3.5.3) of SPSS (version 25.0 for Windows). The general characteristics of participants and study variables were analyzed using descriptive statistics. Pearson’s correlation analysis was used to identify correlations between the study variables. A covariate (confounding variable) related to both mediator and dependent variables may affect mediation effects [26]. Possible covariates were therefore investigated using *t*-test, ANOVA, or Pearson’s correlation analysis from the demographic and medical information. The hypothesized mediation model was analyzed using the PROCESS macro with 10,000 bootstrap bias-corrected 95% confidence intervals (CIs). If a 95% bootstrap CI of a mediation (indirect) effect did not contain zero, it was considered statistically significant [26].

## 3. Results

### 3.1. Participant Characteristics and Study Variables

The mean age of the participants was 60.35 years (SD = 11.19 years). The participants included 64.1% males, 39.4% high-school graduates, 46.9% who earned more than 3,000,000 KRW monthly, and 75.6% who were taking oral hypoglycemic agents as a treatment regimen. Controlled blood glycemia levels (HbA1c < 6.5%) were observed in 25.4%, and the mean diabetes duration was 11.77 years (SD = 8.72 years). Diabetes education had not been experienced by 24.4% of the participants. Table 1 lists the mean scores for health literacy, social isolation, self-efficacy, and self-management.

### 3.2. Correlations among Study Variables

Significant correlations were found between health literacy, self-efficacy, and self-management. Social isolation was negatively correlated with all of the other study variables (Table 2).

### 3.3. Diabetes Education as a Covariate

Participants who had received diabetes education demonstrated significantly lower scores for social isolation (*t* = –3.03, *p* = 0.003) (Table 3) and higher scores for self-efficacy (*t* = 3.54, *p* = 0.001) and self-management (*t* = 4.70, *p* < 0.001). Diabetes education was therefore controlled as a covariate during the mediation analysis. Other demographic and medical information were not significantly associated with both mediation variables (social isolation and self-efficacy) and self-management.

### 3.4. Mediation Model Analysis While Controlling for Diabetes Education

**Figure 1:**
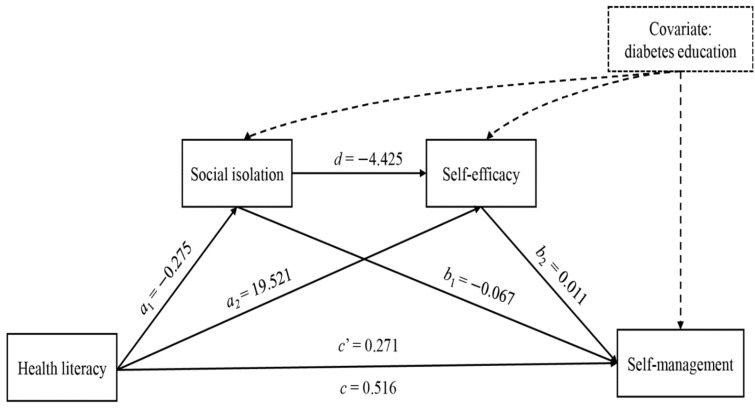
Mediation model linking health literacy with self-management while controlling for diabetes education. *a*_1_, regression coefficient of health literacy predicting social isolation while controlling for diabetes education; *a*_2_, regression coefficient of health literacy predicting self-efficacy while controlling for social isolation and diabetes education; *b*_1_, regression coefficient of social isolation predicting self-management while controlling for health literacy, self-efficacy, and diabetes education; *b*_2_, regression coefficient of self-efficacy predicting self-management while controlling for health literacy, social isolation, and diabetes education; *c*, total effect while controlling for diabetes education; *c*’, regression coefficient of health literacy predicting self-management while controlling for social isolation, self-efficacy, and diabetes education; *d*, regression coefficient of social isolation predicting self-efficacy while controlling for health literacy and diabetes education. All coefficients were significant.

When controlling for diabetes education for the mediation model (Figure 1, Table 4, and Supplementary Table S1), the total effect (*c*) of health literacy on self-management was divided into direct and indirect components. The direct effect (*c*’) was the influence of health literacy on self-management while controlling for all other variables. The three indirect effects were the influence of health literacy on self-management via social isolation only (*a*_1_*b*_1_), self-efficacy only (*a*_2_*b*_2_), and both social isolation and self-efficacy serially (*a*_1_*db*_2_).

The total (*c* = 0.516, 95% bootstrap CI = 0.453–0.580) and direct (*c*’ = 0.271, 95% bootstrap CI = 0.203–0.339) effects of health literacy on self-management were positive and significant, as the CI did not include zero. The mediation model of Figure 1 contained three indirect effects. The first indirect effect of health literacy on self-management via only social isolation while controlling for self-efficacy and diabetes education was estimated as *a*_1_*b*_1_ (–0.275 × –0.067) = 0.018, with a 95% bootstrap CI of 0.004–0.036. This indirect effect was significant, indicating that social isolation mediated health literacy and self-management while controlling for self-efficacy and diabetes education. The second indirect effect of health literacy on self-management via only self-efficacy while controlling for social isolation and diabetes education was significant, and estimated as *a*_2_*b*_2_ (19.521 × 0.011) = 0.214, with a 95% bootstrap CI of 0.165–0.266. Those with greater health literacy therefore had greater self-efficacy, which induces greater self-management, and was independent of social isolation and diabetes education. The third indirect effect of health literacy on self-management via social isolation and self-efficacy serially while controlling for diabetes education was estimated as *a*_1_*db*_2_ (–0.275 × –4.425 × 0.011) = 0.013, with a 95% bootstrap CI of 0.006–0.023, indicating that the indirect effect from these two serial mediators was significant while controlling for diabetes education. The total sum of the indirect effects was also significant, and was estimated as *a*_1_*b*_1_ + *a*_2_*b*_2_ + *a*_1_*db*_2_ (0.018 + 0.214 + 0.013) = 0.245, with a 95% bootstrap CI of 0.196–0.299 (Table 4).

Table 4 lists three pairwise comparisons between the magnitudes of indirect effects while controlling for diabetes education. The magnitude of the indirect effect of health literacy on self-management via social isolation was smaller than that via self-efficacy (95% bootstrap CI = –0.253 to –0.141). The magnitude of the indirect effect via self-efficacy was larger than that via both social isolation and self-efficacy serially (95% bootstrap CI = 0.151–0.253). However, there was no significant difference in the magnitudes of the indirect effects via social isolation only and social isolation and self-efficacy serially (95% bootstrap CI = –0.012 to 0.023).

## 4. Discussion

This study found that health literacy was directly related to self-management, which is consistent with a previous literature review and other empirical studies [5,6]. However, other studies have not found the relationship [32,33,34]. These studies measured health literacy using the Test of Functional Health Literacy in Adults (TOFHLA) [35] or the Rapid Estimate of Adult Literacy in Medicine–Revised (REALM-R) [36], which only assess from a narrow perspective of health literacy (i.e., reading ability and comprehension). Generic instruments such as TOFHLA and REALM-R have been criticized as not comprehensively measuring the skills needed to obtain and use diabetes information when making health decisions [8,10]. A systematic review of 13 health literacy instruments performed on people with diabetes also recommended the use of disease-specific instruments in clinical practice [37]. Considering these recommendations, the contrasting findings might be attributable to the differing instruments used in the studies. Future studies should therefore investigate the direct relationship using a diabetes-specific and comprehensive health literacy instrument, such as the Health Literacy Scale [38] or the DHLS [29].

All three specific indirect mediation pathways were supported by the findings of the present study. The first pathway of health literacy was indicated to have an indirect relationship with self-management via social isolation only. This may be interpreted as people with low health literacy possibly feeling ashamed of or hiding their health literacy ability from others [39], and they will be unable to maintain a social network or frequent contact with others who could be resources to consult and help them with their health problems [18]. Then, social isolation, which refers to decreased social contacts and network sizes, might negatively affect diabetes self-management [23]. The present study has provided the first empirical evidence of the mediating role of social isolation on the relationship between health literacy and diabetes self-management. On top of the quantitative connection of social isolation, a qualitative social connection (e.g., perceived social support) has also been indicated to mediate the relationship between health literacy and diabetes self-management [34]. Further studies are therefore recommended to determine the relationship using quantitative and qualitative social connections of social isolation and perceived social support together as mediators.

The second mediation pathway for health literacy and self-management via self-efficacy was also supported by this study. This finding was concordant with a recent systematic review study that indicated self-efficacy to be a major mediator for the pathways linking health literacy to health behaviors, particularly within self-care of chronic conditions [15]. This finding suggests that health professionals should consider interventions to improve both health literacy and self-efficacy for diabetes self-management in clinical practice.

Lee et al. [40] suggested that enhancing communicative health literacy can effectively improve the self-efficacy of people with type 2 diabetes. If this is the case, using the Diabetes Literacy and Numeracy Education Toolkit—which was developed to facilitate communication between patients and providers to promote effective diabetes learning—is recommended in clinical practice [41]. A “teach-back” spoken communication strategy was suggested to ensure that people with low health literacy understand this education [42]. These interventions using educational material and communication strategies that are sensitive to health literacy may improve the self-efficacy and self-management of people with type 2 diabetes.

According to self-efficacy theory, self-efficacy is derived from four main sources: mastery experiences, vicarious experiences, social persuasion, and physiological/emotional arousal [9]. A previous meta-analysis demonstrated that self-efficacy-focused education interventions based on these four sources improves both the self-efficacy and self-management of people with diabetes [43]. Combining the health-literacy-sensitive intervention and strategy mentioned above with that focused on self-efficacy may have a synergistic effect.

The present study also demonstrated the third indirect pathway of how those with greater health literacy levels contact others more frequently or have closer ties to their social networks, which in turn improves their confidence in performing required actions, and finally improves their likelihood of engaging in diabetes self-management activities. To the best our knowledge, the present study is the first to empirically demonstrate that social isolation and self-efficacy are serial mediators that link health literacy to diabetes self-management.

The magnitude of the indirect effect via self-efficacy was larger than those of two specific indirect effects in this study. This provides a practical direction for health professionals to focus more on the indirect effect via self-efficacy than other indirect effects when planning interventions for the self-management of those with type 2 diabetes.

### Strength and Limitation

This study had theoretical and methodological strengths. The first strength was developing an empirical evidence-based mediation model, which may strengthen explanations of how health literacy affects diabetes self-management. This model may become a theoretical foundation for expanding the health literacy knowledge and its outcomes.

The second strength was the instruments used to measure self-management and self-efficacy. The most frequently used instrument for diabetes self-management is the Summary of Diabetes Self-Care Activities Measure–Revised (SDSCA-R) [44]. However, a recent systematic review of the measurement properties of 13 different diabetes self-management instruments indicated that using the SDSCA-R must be reconsidered due to a lack of psychometric evidence [45]. A new, comprehensive, and psychometrically satisfactory diabetes self-management instrument was used in the present study. To measure self-efficacy, the K-DMSES was used in the present study. A systematic review of 12 self-efficacy instruments used on people with type 2 diabetes indicated that the K-DMSES (particularly the four-factor, 16-item version) was the best, since there is sufficient high-quality evidence for its structural and internal consistency, and sufficient moderate-quality evidence for its reliability and convergent validity [46].

The third strength of the present study was the type of analysis used to determine mediation effects. The mediation model in this study was assessed using the PROCESS macro with bootstrapping to account for the inferential limitations when assuming a normal distribution. This approach had greater statistical power than that proposed by Baron and Kenny [47], and the Sobel test [48]. Structural equation modeling (SEM) is another approach that is increasingly being used in mediation analyses. While standard errors tend to have a downward bias when applying SEM to small samples, SEM has the advantage of accounting for random measurement errors, which is a weakness of the PROCESS macro based on an observed-variable modeling method [49]. However, results are substantially similar when applying SEM and PROCESS to a sufficient sample [49].

A systematic review of empirical studies on the pathway linking health literacy to health behaviors and outcomes suggested that most studies have not controlled for covariates [14]. The fourth strength of the present study was controlling the covariate of diabetes education in order to avoid any threat to the validity of the mediation model.

Notwithstanding the abovementioned strengths, the findings of this study must be cautiously interpreted due to its cross-sectional design, where temporal sequence in the mediation model was difficult to identify. It is therefore suggested to test the mediation model using a longitudinal research design in the future.

## 5. Conclusions

This study demonstrated that health literacy affected diabetes self-management via one direct and three specific-indirect pathways through social isolation only, self-efficacy only, and both social isolation and self-efficacy serially. The magnitude of the indirect effect via self-efficacy was larger than those of the other indirect effects. The mediation model indicated that clinical practice can be improved by providing a more comprehensive self-management intervention to people with diabetes that enhances all the components of health literacy, social contacts/networks, and self-efficacy in particular.

## Figures and Tables

**Table 1:** Descriptive statistics of participants and study variables (*N* = 524).

Variable	Category	*n* (%)	Mean (SD)
Age, years	≤30	7 (1.3)	60.35 (11.19)
	31–40	15 (2.9)	
	41–50	69 (13.2)	
	51–60	163 (31.1)	
	61–70	174 (33.2)	
	≥71	96 (18.3)	
Gender	Male	336 (64.1)	
	Female	188 (35.9)	
Education status	Elementary school	50 (9.5)	
	Middle school	74 (14.1)	
	High school	208 (39.7)	
	College and above	187 (35.7)	
	Others	5 (1.0)	
Monthly income, KRW	≤3,000,000	278 (53.1)	
	>3,000,000	246 (46.9)	
Treatment regimen	OHA	396 (75.6)	
	OHA + insulin	119 (22.7)	
	Insulin	9 (1.7)	
HbA1c	Controlled (<6.5%)	133 (25.4)	7.30 (1.27)
	Uncontrolled (≥6.5%)	391 (74.6)	
Duration of disease, years			11.77 (8.72)
Diabetes education	Yes	396 (75.6)	
	No	128 (24.4)	
Health literacy			2.53 (0.88)
Social isolation			1.07 (1.03)
Self-efficacy			110.45 (32.56)
Self-management			2.20 (0.79)

HbA1c, glycated hemoglobin; KRW, South Korean won; OHA, oral hypoglycemic agents; SD, standard deviation.

**Table 2:** Correlations between health literacy, social isolation, self-efficacy, and self-management (*N* = 524).

Variable	Health Literacy	Social Isolation	Self-Efficacy
Social isolation	–0.25 (*p* < 0.001)		
Self-efficacy	0.57 (*p* < 0.001)	–0.28 (*p* < 0.001)	
Self-management	0.59 (*p* < 0.001)	–0.29 (*p* < 0.001)	0.65 (*p* < 0.001)

**Table 3:** Mean differences in social isolation, self-efficacy, and self-management according to diabetes education.

Study variable	Diabetes Education			
	Yes Mean (SD)	No Mean (SD)	*t*	*p*
Social isolation	0.99 (1.06)	1.31 (1.06)	–3.03 ^a^	0.003
Self-efficacy	113.60 (30.27)	100.74 (37.28)	3.54 ^a^	0.001
Self-management	2.28 (0.78)	1.96 (0.79)	4.70	<0.001

^a^ equal variances were not assumed.

**Table 4:** Total direct, direct, total indirect, and indirect effects, and pairwise comparisons between indirect effects with 95% bootstrap confidence intervals while controlling for diabetes education.

	Product of Coefficient		95% Bootstrap CI	
	Point Estimate	SE	Lower CI Limit	Upper CI Limit
Total effect				
*c*	0.516	0.032	0.453	0.580
Direct effect				
*c*’	0.271	0.034	0.203	0.339
Indirect effects				
*a*_1_ *b*_1_	0.018	0.008	0.004	0.036
*a*_2_ *b*_2_	0.214	0.026	0.165	0.266
*a*_1_ *db*_2_	0.013	0.004	0.006	0.023
Total indirect effects				
*a*_1_*b*_1_ + *a*_2_*b*_2_ + *a*_1_*db*_2_	0.245	0.026	0.196	0.299
Pairwise comparisons between indirect effects				
*a*_1_*b*_1_ − *a*_2_*b*_2_	–0.195	0.028	–0.253	–0.141
*a*_1_*b*_1_ − *a*_1_*db*_2_	0.005	0.009	–0.012	0.023
*a*_2_*b*_2_ − *a*_1_*db*_2_	0.200	0.026	0.151	0.253

*a*_1_*b*_1_, indirect effect of health literacy on self-management via social isolation while controlling for self-efficacy and diabetes education; *a*_2_*b*_2_, indirect effect of health literacy on self-management via self-efficacy while controlling for social isolation and diabetes education; *a*_1_*db*_2_, indirect effect of health literacy on self-management via social isolation and self-efficacy serially while controlling for diabetes education; *a*_1_*b*_1_ + *a*_2_*b*_2_ + *a*_1_*db*_2_, total indirect effect while controlling for diabetes education; *c*, total effect while controlling for diabetes education; *c*’, regression coefficient of health literacy predicting self-management while controlling for social isolation, self-efficacy, and diabetes education. SE, bootstrap standard error.

## Data Availability

The data presented in this study are available on request from the corresponding author. The data are not publicly available due to ethical reasons.

## Supplementary Materials

The following are available online at https://www.mdpi.com/article/10.3390/healthcare9121734/s1, Table S1: Summary information for the mediator model as depicted in Figure 1.

## References

[1] International Diabetes Federation IDF Diabetes Atlas 2019. http://www.diabetesatlas.org.

[2] American Association of Diabetes Educators (2020). An effective model of diabetes care and education: Revising the AADE7 self-care behaviors^®^. Diabetes Educ..

[3] Nutbeam D. (1998). Health promotion glossary. Health Promot. Int..

[4] Paasche-Orlow M.K., Wolf M.S. (2007). The causal pathways linking health literacy to health outcomes. Am. J. Health Behav..

[5] Guo X.M., Zhai X., Hou B.R. (2020). Adequacy of health literacy and its effect on diabetes self-management: A meta-analysis. Aust. J. Prim. Health.

[6] Lee E.H., Lee Y.W., Chae D., Lee K.W., Chung J.O., Hong S., Kim S.H., Kang E.H. (2020). A new self-management scale with a hierarchical structure for patients with type 2 diabetes. Asian Nurs. Res..

[7] Bailey S.C., Brega A.G., Crutchfield T.M., Elasy T., Herr H., Kaphingst K., Karter A.J., Moreland-Russell S., Osborn C.Y., Pignon M. (2014). Update on health literacy and diabetes. Diabetes Educ..

[8] Fransen M.P., von Wagner C., Essink-Bot M.-L. (2012). Diabetes self-management in patients with low health literacy: Ordering findings from literature in a health literacy framework. Patient Educ. Couns..

[9] Bandura A. (1997). Self-Efficacy: The Exercise of Control.

[10] Lee E.H., Lee Y.W., Moon S.H. (2016). A structural equation model linking health literacy to self-efficacy, self-care activities, and health-related quality of life in patients with type 2 diabetes. Asian Nurs. Res..

[11] Huang Y.M., Shiyanbola O.O., Chan H.Y. (2018). A path model linking health literacy, medication self-efficacy, medication adherence, and glycemic control. Patient Educ. Couns..

[12] Al Sayah F., Majumdar S.R., Williams B., Robertson S., Johnson J.A. (2013). Health literacy and health outcomes in diabetes: A systematic review. J. Gen. Intern. Med..

[13] Squiers L., Peinado S., Berkman N., Boudewyns V., McCormack L. (2012). The health literacy skills framework. J. Health Commun..

[14] Bohanny W., Wu S.F.V., Liu C.Y., Yeh S.H., Tsay S.L., Wang T.J. (2013). Health literacy, self-efficacy, and self-care behaviors in patients with type 2 diabetes mellitus. J. Am. Assoc. Nurse Pract..

[15] Cudjoe J., Delva S., Cajita M., Han H.R. (2020). Empirically tested health literacy frameworks. Health Lit. Res. Pract..

[16] Shankar A., McMunn A., Banks J., Steptoe A. (2011). Loneliness, social isolation, and behavioral and biological health indicators in older adults. Health Psychol..

[17] Sentell T.L., Agner J.L., Davis J., Mannem S., Seto T.B., Valente T.W., Varwer M., Taira D.A. (2021). Social networks in patients hospitalized with preventable conditions for heart disease and diabetes in Hawai ‘i by health literacy. Chronic Illn..

[18] Geboers B., Reijneveld S.A., Jansen C.J., de Winter A.F. (2016). Health literacy is associated with health behaviors and social factors among older adults: Results from the lifelines cohort study. J. Health Commun..

[19] Smith S.G., Jackson S.E., Kobayashi L.C., Steptoe A. (2018). Social isolation, health literacy, and mortality risk: Findings from the English longitudinal study of ageing. Health Psychol..

[20] Kobayashi L.C., Steptoe A. (2018). Social isolation, loneliness, and health behaviors at older ages: Longitudinal cohort study. Ann Behav. Med..

[21] Schrempft S., Jackowska M., Hamer M., Steptoe A. (2019). Associations between social isolation, loneliness, and objective physical activity in older men and women. BMC Public Health.

[22] Kadirvelu A., Sadasivan S., Ng S.H. (2012). Social support in type II diabetes care: A case of too little, too late. Diabetes Metab. Syndr. Obes..

[23] Suhl E., Bonsignore P. (2006). Diabetes self-management education for older adults: General principles and practical application. Diabetes Spectr..

[24] Köbling T., Váradi Z., Katona E., Somodi S., Kempler P., Páll D., Zrinyi M. (2020). Predictors of dietary self-efficacy in high glycosylated hemoglobin A1c type 2 diabetic patients. J. Int. Med. Res..

[25] Wu F., Sheng Y. (2019). Social support network, social support, self-efficacy, health-promoting behavior and healthy aging among older adults: A pathway analysis. Arch. Gerontol. Geriatr..

[26] Hayes A.F. (2013). Introduction to Mediation, Moderation, and Conditional Process Analysis: A Regression-Based Approach.

[27] Tofighi D., Kelley K. (2020). Indirect effects in sequential mediation models: Evaluating methods for hypothesis testing and confidence interval formation. Multivar. Behav. Res..

[28] Pieters R. (2017). Meaningful mediation analysis: Plausible causal inference and informative communication. J. Consum. Res..

[29] Lee E.H., Lee Y.W., Lee K.W., Nam M., Kim S.H. (2018). A new comprehensive diabetes health literacy scale: Development and psychometric evaluation. Int. J. Nurs. Stud..

[30] Lee E.H., van der Bijl J., Shortridge-Baggett L.M., Han S.J., Moon S.H. (2015). Psychometric properties of the diabetes management self-efficacy scale in Korean patients with type 2 diabetes. Int. J. Endocrinol..

[31] van der Bijl J., Poelgeest-Eeltink A.V., Shortridge-Baggett L. (1999). The psychometric properties of the diabetes management self-efficacy scale for patients with type 2 diabetes mellitus. J. Adv. Nurs..

[32] Kim S., Love F., Quistberg D.A., Shea J.A. (2004). Association of health literacy with self-management behavior in patients with diabetes. Diabetes Care.

[33] Mancuso J.M. (2010). Impact of health literacy and patient trust on glycemic control in an urban USA population. Nurs. Health Sci..

[34] Osborn C.Y., Bains S.S., Egede L.E. (2010). Health literacy, diabetes self-care, and glycemic control in adults with type 2 diabetes. Diabetes Technol. Ther..

[35] Nurss J., Parker R., Williams M., Baker D. (2001). TOFHLA: Test of Functional Health Literacy in Adults.

[36] Bass P.F., Wilson J.F., Griffith C.H. (2003). A shortened instrument for literacy screening. J. Gen. Intern. Med..

[37] Lee E.H., Kim C.J., Lee J., Moon S.H. (2017). Self-administered health literacy instruments for people with diabetes: Systematic review of measurement properties. J. Adv. Nurs..

[38] Ishikawa H., Takeuchi T., Yano E. (2008). Measuring functional, communicative, and critical health literacy among diabetic patients. Diabetes Care.

[39] Parikh N.S., Parker R.M., Nurss J.R., Baker D.W., Williams M.V. (1996). Shame and health literacy: The unspoken connection. Patient Educ. Couns..

[40] Lee Y.J., Shin S.J., Wang R.H., Lin K.D., Lee Y.L., Wang Y.H. (2016). Pathways of empowerment perceptions, health literacy, self-efficacy, and self-care behaviors to glycemic control in patients with type 2 diabetes mellitus. Patient Educ. Couns..

[41] Wolff K., Cavanaugh K., Malone R., Hawk V., Gregory B.P., Davis D., Wallston K., Rothman R.L. (2009). The diabetes literacy and numeracy education toolkit (DLNET): Materials to facilitate diabetes education and management in patients with low literacy and numeracy skills. Diabetes Educ..

[42] Kim S.H., Lee A. (2016). Health-literacy-sensitive diabetes self-management interventions: A systematic review and meta-analysis. Worldviews Evid.-Based Nurs..

[43] Jiang X., Wang J., Lu Y., Jiang H., Li M. (2019). Self-efficacy-focused education in persons with diabetes: A systematic review and meta-analysis. Psychol. Res. Behav. Manag..

[44] Toobert D.J., Hampson S.E., Glasgow R.E. (2000). The summary of diabetes self-care activities measure: Results from 7 studies and a revised scale. Diabetes Care.

[45] Lee J., Lee E.H., Chae D., Kim C.J. (2020). Patient-reported outcome measures for diabetes self-care: A systematic review of measurement properties. Int. J. Nurs. Stud..

[46] Lee J., Lee E.H., Chae D. (2020). Self-efficacy instruments for type 2 diabetes self-care: A systematic review of measurement properties. J. Adv. Nurs..

[47] Baron R.M., Kenny D.A. (1986). The moderator–mediator variable distinction in social psychological research: Conceptual, strategic, and statistical considerations. J. Pers. Soc. Psychol..

[48] Sobel M.E. (1982). Asymptotic confidence intervals for indirect effects in structural equation models. Sociol. Methodol..

[49] Hayes A.F., Montoya A.K., Rockwood N.J. (2017). The analysis of mechanisms and their contingencies: PROCESS versus structural equation modeling. Australas. Mark. J..

