# Supplementary material for: Pathways Linking Health Literacy to Self-Management in People with Type 2 Diabetes
Source: Healthcare (Basel). 2021 Dec 15;9(12):1734. doi: 10.3390/healthcare9121734 (PMC8701917; doi:10.3390/healthcare9121734)
Supplement: Supplementary file 1 [file healthcare-09-01734-s001.zip › healthcare-1467102-supplementary.pdf]

Supplementary

Table S1. Summary information for the mediator model as depicted in Figure 1.

	Consequent variables														
		**Social isolation**					**Self-efficacy**					**Self-management**			
Antecedent variable		**Coeff.**	**SE**	***t***	***p***		**Coeff.**	**SE**	***t***	***p***		**Coeff.**	**SE**	***t***	***p***
Health literacy	a_1	–0.275	0.050	–5.49	<0.001	a_2	19.521	1.370	14.25	<0.001	c'	0.271	0.034	7.88	<0.001
Social isolation		–	–		–	d	–4.425	1.164	–3.80	<0.001	b_1	–0.067	0.025	–2.67	0.008
Self-efficacy		–	–		–		–	–	–	–	b_2	0.011	0.001	11.72	<0.001
Diabetes education		–0.209	0.103	–2.03	0.043		3.361	2.744	1.23	<.001		0.048	0.058	0.83	0.408
Constant		1.924	0.142	13.58	<0.001		63.162	4.381	14.42	<.001		0.340	0.110	3.08	0.002
		$R^2 = 0.07$					$R^2 = 0.35$					$R^2 = 0.50$			
		$F = 20.19$, $p < 0.001$					$F = 93.28$, $p < 0.001$					$F = 130.70$, $p < 0.001$			

a_1, regression coefficient of health literacy predicting social isolation while controlling for diabetes education; a_2, regression coefficient of health literacy predicting self-efficacy while controlling social isolation and diabetes education; b_1, regression coefficient of social isolation predicting self-management while controlling for health literacy, self-efficacy, and diabetes education; b_2, regression coefficient of self-efficacy predicting self-management while controlling for health literacy, social isolation, and diabetes education; c', regression coefficient of health literacy predicting self-management while controlling for social isolation, self-efficacy, and diabetes education; d, regression coefficient of social isolation predicting self-efficacy while controlling for health literacy and diabetes education. Abbreviate: SE, standard error.
